# Antioxidant Activity of the Essential Oils of Different Parts of *Juniperus excelsa* M. Bieb. subsp. *excelsa* and J. *excelsa* M. Bieb. subsp. *polycarpos* (K. Koch) Takhtajan (Cupressaceae)

**Published:** 2011

**Authors:** Sayyed Ahmad Emami, Bibi Fatemeh Abedindo, Mohammad Hassanzadeh-Khayyat

**Affiliations:** a *Department of Pharmacognosy, School of Pharmacy, Mashhad University of Medical Sciences, Mashhad, 91775-1365, Iran.*; b *Pharmaceutical Sciences Research Center, Department of Medicinal Chemistry, School of Pharmacy, Mashhad University of Medical Sciences, Mashhad, 917751365, Iran.*

**Keywords:** *Juniperus excelsa *subsp. *Excels*, *Juniperus excelsa *subsp. *Polycarpos*, Cupressaceae, Essential oils, Antioxidant activity

## Abstract

The essential oils of branchlets and fruits of *Juniperus excelsa *subsp. *excelsa *and *Juniperus excelsa *subsp. *polycarpos *were examined for their antioxidant activity. The compositions of the essential oils were studied by GC and GC-MS. To evaluation the antioxidants activity of the volatile oils, pure components and positive controls at different concentrations, thin-layer chromatography (TLC) screening methods, diphenylpicrylhydrazyl (DPPH) assay, deoxyribose degradation test and modified deoxyribose degradation test were employed. The results of the present study demonstrate some antioxidant activity for the tested essential oils obtained from various parts of both plants. It indicates that the use of these essential oils, in very low concentrations, may be useful as a natural preservative. However before any final conclusion, it is suggested that the antioxidant activity of these oils should also be evaluated by using lipid solvent system methods.

## Introduction

It is accepted that the free radicals play an important role in the development of tissue damage and implicated in the pathogenesis of many disease (1). Interest has increased considerably in finding naturally occurring antioxidants for use in foods or medicines to replace synthetic antioxidants, especially those of reported being carcinogens (2). It is believed that the preservative effect of many plant species and herbs may consider for the presence of antimicrobial and antioxidant constituents in their tissues. The use of many plants for treatment of various inflammatory diseases suggests that oxidative stress plays a role in human disease and intake of antioxidant might improve human health (3-4). Natural crude drug extracts and biologically active compounds isolated from plant species has been an important sources for treating common infections in developing countries (5-6). However, scientific investigation in order to determine the therapeutic potential of these plants is limited (7).

Essential oils of many plants species have been popular in recent years. The use of many plants for treatment of various inflammatory diseases like rheumatism, fever, diabetes, suggests that oxidative stress plays a role in human disease and intake of antioxidants may improve human health (3). Plant derived antioxidant components such as flavonoids and terpenoids are increasingly aimed as important dietary antioxidant factors (8).There is a strong need for effective antioxidants from natural sources as alternatives of commercial antioxidants. Various researches show that the main compounds of the essential oils have antioxidant activity (9). Essential oils are known to possess potential as natural agents for food preservation. Many of them, recently, have been qualified as natural antioxidants and proposed as potential substitutes of synthetic antioxidants in specific sectors of food preservation where their uses are not in contrast with their aroma (3, 8, 10).

The genus *Juniperus *(Cupressaceae) consists of approximately 67 species and 28 varieties. The genus is divided into three sections: *Caryocedrus *Edlicher (with only one species); *Juniperus *(syn: *Oxycedrus *Spach with 12 species) and *Sabina *(Miller) Spach (with 55 species) (11). Two examined subspecies of Iranian *J. excelsa *subsp. *excelsa *and *J. excelsa *subsp. *polycarpos *belong to the later section (12-13). This study is a part of a systematic investigation on the various aromatic Iranian conifers.

*Juniperus excelsa *M.Bieb. subsp. *excelsa *[*J. sabina *L. var. *taurica *Pall., *J. foetida *var. *excelsa *(M. Bieb.) Spach, *J. isophyllos *K. Koch, *J. excelsa *subsp*. excelsa *var. *depressa *O. Schwarz] is an evergreen tree occasionally a shrub or a prostrate shrub from Cupressaceae which is distributed in Balkan countries, Turkey, Syria and adjacent Lebanon, Georgia, Armenia, Azerbaijan, eastward in Iran to near Ashkhabad in Turkmenia, also on the north east coast of the Black Sea at the foot of the Caucasus and in the Crimea (12-19). Its Persian name is «Arduj» (16, 18-19). *J. excelsa *subsp. *excelsa *is a medicinal plant that has been used to treat dysmenorrheal (18), cough (21), bronchitis and colds (22), jaundice and tuberculosis (23) and to induce menses and expel fetus (24).

*J. excelsa *M.-Bieb. subsp. *polycarpos *(K. Koch) Takhtajan [*J. polycarpos *K. Koch, *J. macropoda *Boiss.] is a dioecious tree to 6-7 m tall or a low shrub with a dense head. This subspecies is found in Afghanistan, Iran, Armenia, Turkey, Turkmenia, India, Uzbekistan, Pakistan, Oman and Saudi Arabia (13, 18, 25). *J. excelsa *subsp. *polycarpos *is a medicinal plant and used for asthma (26); its Persian name is “Ors” (18).

While antioxidant activity of the essential oils of some of the *Juniperus *species has been studied( 3, 27-29), there is no published report on antioxidant activity of the essential oils of *Juniperus excelsa *subsp. *excelsa *and *Juniperus excelsa *subsp. *Polycarpos. *However there is only one published report about the antioxidant activity of *J. excelsa *subsp. *excelsa *(30).

In present study antioxidant effect of the essential oils obtained from different parts *J. excelsa *subsp. *excelsa *and *J. excelsa *subsp. *polycarpos *species evaluated using different methods. Also the compositions of the essential oils of these species analyze using GC and GC-MS in order to determine which components contribute to the antioxidant activity.

## Experimental

*Materials*

Chemicals were obtained from Sigma (Sigma Aldrich GmbH. Steinheim. Germany). Limonene, *β*-pinene, sabinene, *α*-pinene, *α*-thujene, cedrol, *δ*-2-carene, *δ*-3-carene and *γ*-terpinene were purchased from Roth (Karlsruhe, Germany). Thin-layer chromatography (TLC) was carried out using silica gel F_254_ aluminum sheets (Merck, Darmstadt. Germany).

*Plant material*

Different parts of *Juniperus excelsa *subsp. *excelsa *(fruits and leaves) were collected from Eslami Island (1800 m altitude, Nov. 2006), East Azerbaijan province, and north west of Iran. The leaves (male and female trees) and fruits of *J. excelsa *subsp*. polycarpos *were collected from Teel Abad (2000 m altitude, Dec. 2006), Tash region, Khosh yeilagh area, Semnan province, central area of Iran. These plants were identified by Mr. M. R. Joharchi from Ferdowsi University of Mashhad Herbarium (FUMH) where vouchers specimens of both *J. excelsa *subsp. *excelsa *and *J. excelsa *subsp*. polycarpos *are deposited. The herbaria are 97-1005-8 and 97-1005-7 respectively. The collected materials were stored at -20°C in order to avoid unfavorable changes in chemical components (31).

*Isolation of the essential oil*

Defrosted fresh leaves of male and female plants (800 g fresh wt.) as well as fruits (400 g fresh wt.) were cut into small pieces and then ground with a commercial blender. The volatile oils were isolated through distilled steam using a manufactured apparatus with a condenser. Distillation was continued for about 4 h and the volatile compounds containing the water-soluble fraction were allowed to settle for 30 min (32). The essential oils were separated from the aqueous layer and dried over anhydrous sodium sulfate. The dried oils were stored under nitrogen gas in a sealed vial and at -20°C until being analyzed. The yield percentage and composition of the essential oils were expressed in mL/100 g of fresh plant materials.

*GC and GC-MS analysis*

The composition of the volatile oil samples obtained from the fruits and leaves of *J. excelsa *subsp. *excelsa *as well as the fruits and leaves of the male and female of *J. excelsa *subsp*. polycarpos *were identified using gas chromatography (GC) and gas chromatography-mass spectrometric (GC-MS) analysis.

The GC-MS apparatus was a Varian GC-MS spectrometer consisted of a Varian star 3400 gas chromatograph equipped with a fused-silica column (DB-5, 30 m × 0.32 mm i.d., film thickness of 0.25 mμ; J and W Scientific Inc), interfaced with a mass spectrometric detector (Varian Saturn 3). The operating conditions were as follows: oven temperature of 60-240°C with the rate of 3°C/min; injector temperature of 280°C; injector mode: split injection with split ratio of 1 : 20; with the carrier gas, He; flow rate of 2 mL/min; electronic impact (EI), ionization potential 70 eV, ion source temperature of 250°C, ionization current of 1000 μA, resolution of 1000 and mass range of 40-300 u.

The gas chromatograph (GC) was a Shimadzu GC-17 equipped with a FID detector, fused-silica column (DB-5, 25 m × 0.25 mm i.d., film thickness of 0.25 μm). The operating conditions were as follows: oven temperature of 60-280°C with the rate of 8°C /min; injector temperature of 280°C, split ratio of 1 : 10, with the carrier gas, N_2_; detector temperature of 300°C.

The oil components were identified from their retention indices (RI) obtained with reference to *n*-alkane series (Sigma, UK), on DB-5 column, mass spectra with those of authentic samples, composition of their mass spectra and fragmentation patters reported in literature, computer matching with MS-data bank (Saturn version 4). Quantification of the relative amount of the individual components was performed according to the Area Percentage Method without consideration of calibration factor (33).

*Antioxidative assay*

*Rapid TLC screening for antioxidant*

This method was used to evaluate the antioxidant activity of the essential oils, as well as the pure standard compounds and positive controls. Eight pure components (*α*-pinene, sabinene, *β*-pinene, limonene, *δ*-3-carene, *δ*-2-carene, *γ*-terpinene and cedrol, as pure standard components), three different compounds [vitamin C, butylated hydroxytoluene (BHT) and quercetin] as positive controls and the oils obtained from different parts of the plants were spotted on duplicate silica gel plates and developed in toluene-ethyl acetate (97: 3 v/v).

One of the developed TLC plates was sprayed with *β*-carotene-linoleic acid reagent (34). Active compounds were detected as yellow spots on a white background zones where the colors that changed within 30-60 min (after spraying), were taken as positive results. The second developed TLC plate was sprayed with a 0.2% solution of the stable radical diphenylpicrylhydrazyl (DPPH) (35-36). Active compounds were detected as yellow spots on a purple background in these TLC plates. Zones in which the color changed within 30 min (after spraying) were taken as positive results. In both cases, some potential radical scavenging compounds (vitamin C, quercetin) were used as positive controls.

*DPPH free radical scavenging activity*

DPPH method was employed to investigate the antioxidant activity of the oils and their main active components. In this spectrophotometric method, DPPH was used as a reagent in order to 

measure DPPH free radical scavenging activity (35-36)**. **Various concentrations (50 μL) of each volatile oil obtained from different parts of plants, a number of main components of the essential oils compounds (*α*-pinene, sabinene, *β*-pinene, limonene, *δ*-3-carene, *δ*-2-carene, *γ*-terpinene and cedrol) and five different compounds, (vitamin C, vitamin E, dimethyl sulfoxide (DMSO), butylated hydroxytoluene (BHT) and quercetin – as positive controls) were added to 2.5 mL of a 0.004% solution of DPPH in methanol. The reaction mixture was shaken and then incubated for 30 min at room temperature. The amount of DPPH remaining was determined at 517 nm against a blank using a spectrophotometer (Milton Roy Company Spectronic 2OD). All tests were carried out for five times.

*Deoxyribose degradation assay*

The procedure described was used to evaluate the hydroxyl group free radical scavenging activity of samples. In this method, 100 μL of 28 mM 2-deoxy-2-ribose in phosphate buffer (pH = 7.4), 500 μL of solutions from various concentrations of the volatile oil obtained from different parts of the plants or the other main components of the essential oils compounds (*α*-pinene, sabinene, *β*-pinene, limonene, *δ*-3-carene, *δ*-2-carene, *γ*-terpinene and cedrol), as well as quercetin and dimethyl sulfoxide (DMSO) (in phosphate buffer containing 1.5% of Tween 80), 200 μL of mixture of 1.04 mM EDTA and 200 μM FeCl3 (1:1 v/v), 100 μL of 1.0 mM H_2_O_2 _and 100 μL of 1.0 mM ascorbic acid were mixed. All solutions were prepared freshly. The reaction mixtures (final volume = 1.0 mL) were incubated for 1 h at 37°C. One milliliter of thiobarbituric acid (TBA) (1%) and 1.0 mL of trichloroacetic acid (TCA) (2.8%) were added to each of these incubated samples and again incubated for 20 min at 100˚C. After cooling, at 532 nm, the absorbance of the samples was measured against a blank containing deoxyribose and buffer. Reactions were carried out for five times. The inhibition percent (I) of deoxyribose degradation was calculated in the following way:

I (%) = 100 × (A_0_ - A_1_ / A_0_)

Here, A_0_ is the absorbance of the control reaction (reaction, containing no test compound) and A_1_ is the absorbance of the test compound (3, 10).

*Assay for site-specific actions*

Several tests were performed in order to assess possible site-specific actions which only present another feature of deoxyribose method (37-38)**. **

Performing these tests indicates whether the oils possess pro-oxidative activities, if they can stimulate an oxidative process or if they generate fragments, which react with thiobarbituric acid. The described deoxyribose method was adapted in three ways:

1. FeCl_3_ was used instead of a solution of Fe ^3+^- EDTA

2. Ascorbic acid was omitted from the test system

3. The reaction was performed without deoxyribose. Phosphate buffer with pH of 7.4 was used to compensate the lacking volume. All experiments were carried out in triplicate (3).

*Statistical analysis*

Values expressed are mean ± SD. All statistical analyses were carried out using SPSS 11.5 for windows.

## Results and Discussion

*GC-MS analysis*

All the volatile oil samples obtained from the fruits and leaves of *J. excelsa *subsp. *excelsa *as well as the fruits and leaves of male and female of *J. excelsa *subsp*. polycarpos *were clear and possessed a strong odor. The essential oils isolated from leaves of female and fruits of *J. excelsa *subsp. *polycarpos *were colorless and yielded 1.00% and 1.12% (v/w) of volatile oil respectively, while leaves of male *J. excelsa *subsp. *polycarpos *yielded 0.62% of pale yellow essential oils. The volatile oils isolated separately from fruits and leaves of *J. excelsa *subsp. *excelsa *were light yellow and colorless, yielded 1.66% and 1.50% (v/w) of volatile oil respectively. All the obtained essential oils were analyzed through GC and GC-MS. 

The constituents of the essential oils of *J. excelsa *subsp. *polycarpos *and *J. excelsa *subsp. *excelsa *are listed in Tables 1 and 2 respectively in order of elution from the DB5 column. In the volatile oils obtained from leaves of both male and female *J. excelsa *subsp. *polycarpos, *27 and 33 compounds (representing more than 98.15% and 92.54% of the total essential oil compounds respectively) and in the oil of fruits of this plant, 24 compounds (representing more than 98.34% of the total essential oil compounds) were identified (Table 1).

**Table 1:** Chemical composition of the volatile oil from male and female leaves of *Juniperus excelsa *subsp*. polycarpos*

**Components **	**Retention Index** ^(1)^		**Male Leaves%**	**Female Leaves%**		**Fruits%**
*α *-Pinene	938		32.72	59.90		78.26
Sabinene	977		3.57	t		1.51
*ß *-Pinene	984		15.83	2.63		t
Myrcene	995		0.10	0.12		0.22
*δ*-2-Carene	1000		0.22	2.34		3.30
*α *-Phellandrene	1007		2.90	t		t
1,4-Cineol	1012		6.50	6.79		6.97
*α *-Terpinene	1020		0.12	0.56		t
Cymene (para)	1023		0.25	-		-
Limonene	1031		7.02	9.73		0.21
Z-*ß*-Ocimene	1039		0.10	-		t
E-*ß*-Ocimene	1047		1.82	5.54		3.48
Bergamal	1057		5.05	0.11		-
*γ *-Terpinene	1064		0.20	0.75		t
Sabinene hydrate (*cis*)	1069		0.55	t		-
Cymenene (meta)	1087		1.03	-		-
6-Camphenol	1112		2.28	2.08		1.87
*α *-Campholenal	1125		3.53	3.29		t
Ocimene (allo-)	1129		0.54	-		-
Limonene oxide (*cis*)	1136		0.52	0.13		t
Borneol	1165		0.46	0.61		0.32
3-Thujanol	1172		0.55	0.10		0.13
*α *-Terpineol	1191		0.13	0.10		0.19
Verbenone	1207		0.12	t		-
Z-Ocimenone	1231		0.10	0.10		t
E-Caryophyllene	1417		-	-		0.18
γ- Elemene	1435		0.55	0.85		0.21
E-β -Farnesene	1459		0.45	1.51		-
Germacrene D	1482		0.10	-		0.18
α-Muurolene	1498		0.13	0.58		-
Cuparene	1507		-	0.13		-
γ- Cadinene	1516		-	0.13		-
α -Cadinene	1541		0.16	0.50		t
Widdrol	1597		-	0.12		-
Cedrol	1621		1.88	2.57		1.31
Grouped compounds:						
Monoterpene hydrocarbons		81.57		66.42		86.98
Oxygen-containing monoterpenes		13.31		19.73		9.48
Sesquiterpene hydrocarbons		1.39		3.70		0.57
Oxygen-containing sesquiterpenes		1.88			2.69	1.31	

t: trace (< 0.1%) ; (1): The retention Kovats indices were determined on DB-5 capillary column

 Monoterpene hydrocarbons were the main constituents of these essential oils (86.98, 81.57 and 66.42% for the oil of fruits and leaves of male and female trees respectively). The analysis also indicated that the amounts of sesquiterpene hydrocarbons and oxygen-containing sesquiterpenoids were low in the oils of fruits and leaves of male and female trees of *J. excelsa *subsp*. polycarpos, *while the amounts of oxygen-containing monoterpenoids were relatively high (9.48, 13.31 and 19.73% respectively) in these oils.

The main compounds in the oils of both male and female leaves as well as the fruits of this plant were *α*-pinene (59.90, 32.72, and 78.26%), 1,4-cineol (6.79, 6.50 and 6.97%) and limonene (9.73, 7.02 and 0.21%) respectively. However *β*-pinene (15.83%) was also one of the major components of *J. excelsa *subsp*. polycarpos *leaves of female tree oil.

The analysis of the leaves’ and fruits’ oil of *J. excelsa *subsp. *excelsa *essential oils leads to identifying 39 and 35 compounds respectively (representing more than 90.15% and 94.35% of the total essential oil compounds respectively) (Table 2). 

**Table 2:** Chemical composition of the volatile oil from fruits and leaves of *Juniperus excelsa *subsp. *excels*

**Components **	**Retention Index** ^(1)^	**Leaves%**	**Fruits%**
*α *-Pinene	940	32.34	47.64
Camphene	952	0.92	t
Verbena	967	0.10	t
Sabinene	973	0.39	-
*β*-Pinene	983	2.20	2.71
Myrcene	994	5.40	5.91
*δ*-2-Carene	1004	2.80	1.25
*α *-Terpinene	1016	0.12	0.13
Cymene (para)	1022	0.42	-
Limonene	1032	4.40	4.50
*γ *-Terpinene	1065	0.90	1.12
Terpinolene	1091	2.85	1.76
*α *-Campholenal	1128	0.28	-
E-Pinocarveol	1140	0.20	-
Camphor	1148	0.72	0.21
Pinocarvone	1164	t	t
Borneol	1167	0.14	-
Pinocamphone(*cis*)	1175	0.24	0.16
Terpinene-4-ol	1182	-	0.13
Verbenone	1204	0.97	t
Bornyl acetate	1286	t	0.92
*δ*-Elemene	1339	0.11	0.54
*α *-Copaene	1375	0.13	t
*β*- Elemene	1388	3.74	0.43
Z-Caryophyllene	1407	2.60	1.1
*β*-Caryophyllene	1422	2.20	3.60
*γ*- Elemene	1434	2.80	5.50
*α*-Humulene	1453	0.67	t
E-*β *-Farnesene	1460	0.34	0.44
Germacrene D	1483	0.40	0.92
*α *-Muurolene	1497	0.36	0.18
Z-*α *-Bisabolene	1508	2.91	0.18
*γ*-Cadinene	1516	2.22	t
*δ*-Cadinene	1524	0.79	0.76
E-*γ*-Bisabolene	1533	0.56	0.65
*α *-Cadinene	1542	0.40	t
Elemol	1553	0.34	0.55
Germacrene B	1564	0.60	0.42
*α *-Cedrol	1604	13.06	12.01
*α-*Cadinol	1655	0.53	0.63
Grouped compounds:			
Monoterpene hydrocarbons		53.56	65.23
Oxygen-containing monoterpenes		1.83	1.21
Sesquiterpene hydrocarbons		20.83	14.72
Oxygen-containing sesquiterpenes		13.93	13.19

t: trace (< 0.1%) ; Retention Index: The retention Kovats indices were determined on DB-5 capillary column

The major components in the oils of *J. excelsa *subsp. *excelsa *leaves and fruits were monoterpene hydrocarbons (53.56% and 65.23%), sesquiterpene hydrocarbons (20.83% and 14.72%) and oxygen-containing sesquiterpenes (13.93% and 13.19%), respectively. However, the amounts of oxygen-containing monoterpenes were low in the analyzed oils. The main constituents of the leaves’ and fruits’ oils of *J. excelsa *subsp. *excelsa *were *α*-pinene (32.34% and 47.64%), *α*- cedrol (13.06% and 12.01%) and *β*-myrcene (5.40% and 5.91%) respectively.

Several reports published about the main constituents of the leaves’ and fruits’ oils obtained from *J. excelsa *subsp. *polycarpos *and *J. excelsa *subsp. *excels *(39-43).

The results of this study did not entirely correspond with the published data. The comparison of the numbers and amounts of components in the essential oils of these plants grown in different parts of the world indicated that the oil composition of individual plants may vary widely due to the climate, growing area, time of collection, *etc*., and these differences are very common (44).

*Antioxidative assay*

*Rapid TLC screening*

In rapid TLC screening test, the essential oils of different parts of the plants, their pure components and positive controls were tested for their antioxidant activity.

One of the developed TLC plates (in duplicate) containing the tested compounds was sprayed with *β*-carotene – linoleic acid reagent. Another developed TLC plate (in duplicate) with the tested compounds was sprayed using a solution of DPPH. When the TLC plate was sprayed with *β*-carotene-linoleic acid reagent, only one yellow zone related to the oil was detectable for each essential oil. In both tests, all pure compounds produced yellow spots. Various monoterpene hydrocarbons have very similar polarity and similar retention indices; therefore they accumulate on the same area of the plate. This may explain why only one yellow zone (related to a group of compound) appeared for each one of the tested oils and explain the incomplete separation on the TLC plates. In this test, all the volatile oils – pure standard components and positive controls–showed some antioxidant activity. Considering the results obtained from using TLC screening method to evaluate the antioxidant activity of the essential oils obtained from both *J. excelsa *subsp. *polycarpos *and *J. excelsa *subsp. *excelsa *indicates that the TLC method can be used as a rapid test to detect antioxidant effects of samples, but it is not appropriate to identify which compounds in the oil correspond to the antioxidant effect. Therefore, the oils and other pure components that possessed antioxidant activity were subjected to further testing.

*DPPH free radical scavenging activity*

The abilities of the test compounds (both the essential oils and their main components) to donate hydrogen atoms or electrons were measured spectrophotometrically in DPPH assay. The testing materials which reduced DPPH to the yellow colored product–diphenylpicrylhydrazine– and decreased the absorbance at 517 nm, possessed antioxidant activity.

In this experiment, pure compounds used as standard showed very different antioxidant activity ranged from 17.7 (in concentration of 4μL/mL) for *γ*-terpinene, no activity for *α*-pinene and limonene and very low activity for *β*-pinene. For the compounds used as positive controls, while DMSO and vitamin E showed very low activity, quercetin and ascorbic acid possessed relatively high antioxidant effects (77.7 and 38.7% respectively) (Table 3).

**Table 3:** Antioxidant activity (%) of the volatile oils obtained from various parts of *Juniperus excelsa *subsp*. polycarpos *and *J. excelsa *subsp. *excels *as well as other tested compounds in DPPH assay

**Concentration (μL/mL)**					
**4**	**2**	**1**	**0.5**	**0.1**	
77.7(3.2)	37.9 (1.6)	21.8 (0.5)	10.6 (0.8)	4.2 (0.4) ^(1)^	Quercetin
38.7(1.0)	19.3 (0.3)	11.1 (1.8)	4.9 (0.3)	1.3	Vitamin C
13.3(3.6)	4.8 (0.6)	2.6 (0.4)	1.9 (0.3)	0.5	BHT
2.4(0.9)	0.7 (0.3)	1.0 (0.4)	0.3 (0.2)	0.6 (0.4)	DMSO
5.26(0.32)	1.10 (1.1)	NA	NA	NA	Vitamin E
17.7(0.3)	8.5 (0.6)	5.6 (0.4)	3.3 (0.4)	0.8 (0.4)	*γ*-Terpinene
3.1(0.4)	1.5 (0.342)	1.8 (0.4)	1.6 (0.3)	0.8 (0.3)	Cedrol
13.0(3.6)	4.8 (0.6)	2.3 (0.4)	1.9 (0.3)	0.5	*δ*-2-Carene
4.8(0.4)	1.1 (0.6)	0.5 (0.2)	0.2 (0.1)	0.2 (0.1)	*δ*-3-Carene
NA	0.4 (0.2)	2.1 (0.3)	NA	0.2 (0.2)	Limonene
1.0 (0.4)	0.1 (0.2)	NA	NA	0.4(0.3)	*β*-Pinene
NA	NA	NA	NA	NA	*α*-Pinene
5.1(0.5)	1.8 (0.1)	1.0 (0.4)	1.4 (0.4)	NA	Sabinene
9.8 (0.4)	4.5 (0.5)	5.0 (1.8)	3.5	1.6 (0.7)	*J. excelsa *subsp. *polycarpos *ML
16.8 (0.3)	7.3 (0.4)	5.4 (1.1)	3.2 (0.4)	1.7 (0.3)	*J. excelsa *subsp. *polycarpos *FL
1.4	NA	NA	NA	NA	*J. excelsa *subsp. *polycarpos *FT
8.2(0.6)	4.8 (0.4)	3.0 (0.4)	1.9 (0.5)	1.9 (0.8)	*J. excelsa *subsp. *excels *L
6.6(1.8)	5.1	2.7	1.6 (0.4)	3.8 (0.7)	*J. excelsa *subsp. *excels *FT

(1): Amounts in parenthesis are representing standard deviation; NA: Not Active; ML: Male Leaves, FL: Female Leaves, FT: Fruit, L: Leaves

For the *J. excelsa *subsp. *polycarpos *oils, the strongest effect was measured for the oil of female leaves of the plant in concentration of 4 μL/mL (16.8%) and the weakest effect was related to the oil of *J. excelsa *subsp. *polycarpos *fruits (1.4%).

The leaf and fruit oils of *J. excelsa *subsp. *excelsa *showed low antioxidant activity at the concentration of 4 μL/mL (8.2% and 6.6% respectively), (Table 3). Although the composition of the leaves and fruits oil of *J. excelsa *subsp. *excelsa *is different, their antioxidant activity in this DPPH test are nearly similar (8.2% and 6.6% in concentration of 4 μL/mL respectively). So, one can attribute that each minor or major compound has specific antioxidant activity, but despite the differences in the numbers and percentages of the compounds in these essential oils, the sum or average of this activities are similar.

However, the low antioxidant activity of all the examined oils in DPPH test may be partially due to the various amounts of inactive compounds in DPPH test (*e.g*. low amounts of *γ*- terpinene as well as high amounts of *α*-pinene), (Tables 1 and 2).

Besides, it is likely that the low activity of all the tested essential oils in this test is due to its unknown components or because of some compounds that exist in trace amounts and did not subject to the antioxidant tests (3).

*Deoxyribose degradation assay*

In deoxyribose degradation assay, the ability of a compound to remove hydroxyl radical and prevent sugar from degradation was tested. Most of the tested compounds showed some antioxidant effects. In deoxyribose degradation test, OH radicals were generated by the reaction of ferric-EDTA together with H_2_O_2 _and ascorbic acid to attack the substrate deoxyribose. The resulting products of the radical attack formed a pink chromogen when it was heated with TBA in acid solution. Incubation of this reaction mixture with antioxidant substances made it possible to interfere with the free radical reaction and could prevent damage to the sugar (1, 3, 10, 45).

Quercetin and DMSO as positive controls, showed the highest activity on scavenging OH radicals (44.1 and 55.8 respectively) in this test. The activities of pure standard compounds varied from the highest activity for *β*-pinene, extremely weak antioxidant effects for some other compounds like *α*-pinene and sabinene, and no antioxidant activity for *δ*-2-carene (Table 4).

**Table 4:** Antioxidant activity (%) of the volatile oils obtained from various parts of *Juniperus excelsa *subsp*. polycarpos *and *J. excelsa *subsp. *excels *as well as other tested compounds in deoxyribose assay

**Concentration (μL/mL)**						
**1**	**0.5**	**0.2**	**0.1**	**0.05**		
44.1 (0.9)	33.4 (0.5)	26.0 (0.7)	14.6 (0.5)	3.4 (0.4) ^(1)^	Quercetin	
55.8 (0.7)	44.4 (0.9)	25.3 (1.3)	13.1 (0.4)	7.1 (0.9)	DMSO	
17.1 (1.2)	7.0 (1.5)	12.6 (0.6)	12.2 (0.5)	8.1 (0.5)	*γ*-Terpinene	
26.8 (0.4)	NA	NA	NA	NA	Cedrol	
NA	8.9 (1.0)	2.7 (0.6)	4.9 (0.8)	NA	*δ*-2-Carene	
27.3 (1.0)	29.8 (0.8)	19.0 (1.1)	15.5 (0.5)	12.5 (0.8)	*δ*-3-Carene	
31.7 (1.5)	25.2 (3.4)	34.6 (1.4)	39.5 (1.0)	47.6 (1.4)	*β*-Pinene	
12.6 (2.3)	26.0 (0.9)	40.6 (2.9)	16.3 (1.0)	40.2 (1.2)	Limonene	
4.1 (1.2)	5.8 (0.8)	1.2 (0.4)	NA	NA	α-Pinene	
1.3 (1.4)	8.2 (1.5)	17.0 (1.4)	12.2 (0.8)	NA	Sabinene	
NA	NA	NA	0.3 (0.2)	7.1 (2.8)	*J. excelsa *subsp. *polycarpos *ML	
NA	NA	NA	NA	NA	*J. excelsa *subsp. *polycarpos *FL	
NA	NA	NA	NA	NA	*J. excelsa *subsp. *polycarpos *FT	
8.6 (3.6)	11.2 (3.1)	12.0 (0.8)	9.9 (1.5)	17.1 (5.8)	*J. excelsa *subsp. *excels *L	
3.9 (0.9)	29.5 (5.2)	35.5 (17.4)	31.6 (9.6)	20.8 (1.0)	*J. excelsa *subsp. *polycarpos *ML	

(1): Amounts in parenthesis are representing standard deviation; NA: Not Active ; ML: Male Leaves ; FL: Female Leaves ; FT: Fruit ; L: Leaves

None of the tested volatile oils possessed remarkable antioxidant activity. Maximum inhibition among the essential oils was measured for fruits of *J. excelsa *subs. *excelsa *at 0.2 μL/mL concentration (35.5%). The variability in antioxidant activity of the tested oils can mainly be related to the variability in the amounts of compounds and their specific activity in deoxyribose degradation assay. During this test, antioxidant effects were occurred in some of the oils and pure standard compounds. But at higher concentration, the absorbance increased, the antioxidant effect decreased and sometimes pro-oxidant effect appeared. This may be due to pro-oxidative effect of certain compounds such as alkanals and other aliphatic aldehydes that react with the reagent TBA and form colored products (3, 10, 46). These pro-oxidative effects will be examined in the assay of site-specific action.

*Assay for site-specific actions*

In site-specific reaction assay, the deoxyribose assay was modified in three different ways to assess whether the oils exhibited site-specific effects. In one occasion, the EDTA was omitted from the reaction mixture. Iron was added as ferric chloride instead of complex form of Fe^3+^-EDTA. Some of the Fe^3+^ ions bind directly to the sugar and its degradation becomes site-specific (36). The formed hydroxyl radicals attacked deoxyribose immediately. An inhibition of this degradation in the absence of EDTA depends not only on a scavenger’s ability to react with OH, but also on its potential to form complex with iron ions. None of the test compounds showed remarkable differences when EDTA was omitted.

In another test, the ascorbate was omitted from the reaction mixture in order to examine the ability of a substance to reduce Fe^3+^-EDTA and decrease the rate of OH radical generation. If an agent possesses pro-oxidant activity, the deoxyribose degradation will be stimulated, more fragments will be produced and the absorbance at 532 nm will be increased significantly (10). From all tested samples, all the oils obtained from different parts of *J. excelsa *subsp. *polycarpos *and the oil obtained from fruits of *J. excelsa *subsp. *excels *as well as standard compounds γ-terpinene, limonene and sabinene (l μL/mL) could induce the radical generation. In the presence of TBA at low pH, while yellow chromogens are generated soon after mixing the aldehyde with TBA, red pigments appear about 6 h after the beginning of the reaction. 

In another experiment, deoxyribose was omitted from the reaction mixture in order to see whether the compounds under examination themselves could form degradation products which react with TBA to make chromogen. The omission of deoxyribose from the reaction mixture leaves the tested oils as the only substrates to react with OH radicals and form TBA reactive species (TBARS) (3). The essential oils obtained from fruits and leaves of male *J. excelsa *subsp. *polycarpos *as well as standard compounds like *α*-pinene and limonene were the only substrates to react with OH radicals and to form TBARS. Therefore, the antioxidant activity strength observed for these tested oils and compounds may not be their actual antioxidant activity. The increase of absorption in their solutions may be due to the production of chromogens by various compounds in the solution and therefore, cause a false decrease in antioxidant activity.

## Conclusion

Although the results of present study demonstrate relatively low antioxidant activity for the tested essential oils obtained from various parts of both *J. excelsa *subsp. *polycarpos *and *J. excelsa *subsp. *excels *species, these activities suggest possible use of the essential oils of these two plants in very low concentrations for preserving food materials. However before any final conclusion, it is suggested that these oils’ antioxidant activity should also be evaluated using lipid solvent system methods.
